# Gender and Age Estimation Methods Based on Speech Using Deep Neural Networks

**DOI:** 10.3390/s21144785

**Published:** 2021-07-13

**Authors:** Damian Kwasny, Daria Hemmerling

**Affiliations:** Department of Measurement and Electronics, AGH University of Science and Technology, 30-059 Krakow, Poland; damiankwasny95@gmail.com

**Keywords:** speech processing, neural networks, gender classification, age estimation, x-vector

## Abstract

The speech signal contains a vast spectrum of information about the speaker such as speakers’ gender, age, accent, or health state. In this paper, we explored different approaches to automatic speaker’s gender classification and age estimation system using speech signals. We applied various Deep Neural Network-based embedder architectures such as x-vector and d-vector to age estimation and gender classification tasks. Furthermore, we have applied a transfer learning-based training scheme with pre-training the embedder network for a speaker recognition task using the Vox-Celeb1 dataset and then fine-tuning it for the joint age estimation and gender classification task. The best performing system achieves new state-of-the-art results on the age estimation task using popular TIMIT dataset with a mean absolute error (MAE) of 5.12 years for male and 5.29 years for female speakers and a root-mean square error (RMSE) of 7.24 and 8.12 years for male and female speakers, respectively, and an overall gender recognition accuracy of 99.60%.

## 1. Introduction

Speech is a multidimensional phenomenon, the production of which consists of many anatomical structures movements that influence the overall speech quality and voice characteristics. Speech is the main and the easiest source of communication. Beside the lingustic information it also covers speaker-dependent para-linguistic data such as speaker’s identity, emotional state, health state, age, or gender [1,2]. The systems for automatic extraction of these information from speech might be very useful in numerous applications such as in personal identification in banking systems; customer care applications such as call centers; voice bots; and interactive, intelligent voice assistants. In the industry, there are already global and local companies offering such services for speech processing, like Google, Amazon, and Techmo on polish market [3]. Extracting information about age and gender of the speaker may be used by the interactive voice response system (IVR) to redirect the speaker to an appropriate consultant [4] or to play a suitable for a given gender/age group background music [5]. For voice-bots systems, extraction of para-linguistic information may be applied to alter the behaviour of the bot. In the case of voice assistants, such knowledge may be used to target suitable advertisements or select search results that are more fitting for a given age/gender group. All combined, exploiting the para-linguistic content can lead to an improved user experience, and this in turn may generate revenue for the company that decides to use such systems.

### Related Works

The research on extracting this para-linguistic content has been rapidly explored in the recent years in many languages. In the earlier years, researchers used methods involving extraction of some acoustic parameters such as mel frequency cepstral coefficients (MFCCs) and perceptual linear prediction, which were then averaged across the sequence to form features which were then used in some classification algorithm such as support vector machine (SVM) [5]. Later, an approach based on the idea of embedding the variable-length utterance into a fixed sized embedding vector and applying this vector in an external classifier, such as probabilistic linear discriminant analysis (PLDA) has emerged [6,7]. Before the deep learning (DL) era, the most popular embedding methods were based on so-called i-vectors [6]. In this framework, a universal background model (UBM) and a projection matrix are learned and then used in a PLDA classifier [6].

The authors of [8] present a new deep neural network (DNN)-based embedding framework called x-vector. They trained the time-delay neural network (TDNN) for a speaker classification task. The results in PLDA classifier. The work in [9] presents the implementation of this method on the speaker in the wild (SITW) dataset and in the end the results were better than using i-vector baseline. In [7], the authors applied different DNN-based speech embedding methods on NIST Speaker Recognition Evaluation 2018 (SRE18) [7]. Their results outperform again the i-vector baseline.

Another embedding approach has been introduced by the authors of [10] in the context of a similar task of speaker verification and is called a d-vector. Similarly to the one presented in [8], the authors also embed a variable-length utterance into a fixed-size embedding vector. This methods differs from x-vector in the way the embedding vector is generated: while in the case of x-vector system statistical pooling is performed on the output of the last hidden layer of a convolutional neural network (CNN) to aggregate global context, d-vector architecture is based on a simple multi-layer, long-short time memory (LSTM) recurrent neural network (RNN) and the output of the last cell in the last hidden layer is used as the embedding.

The Multilayer Perceptron (MLP)-based deep learning model for deploying a gender-based user classification was performed in [11]. The proposed model was trained with different set of parameters and finally came up with an MLP model that achieves an accuracy of 96% on the test dataset. In [12], a gender recognition model using IViE corpus dataset was applied with implementation of multiple classifiers including MLP, GMM, Vector quantization, and Learning vector quantization. They obtained 96.4% accuracy from their proposed model. The work in [13] presents a multilayer perceptron deep learning model was applied using the acoustic properties of the voices and speech to identify the gender. The classification model managed to achieve 96.74% accuracy. Alhussein et al. [14] proposed a method that extracted a new type of time-domain acoustic feature for gender detection. Besides, this acoustic feature measured the voice intensity by calculating the area under the modified voice from two different databases to make a difference between males and females. They obtained 98.27% accuracy for clean speech and 96.55% accuracy for noisy speech. The authors of [15] performed data preprocessing to get the noise-free smooth data and used a multi-layer architecture model to extract the features. The experiments were done on three different datasets: TIMIT, RAVDESS, and BGC (Self-Created). They acquired the highest 96.8% accuracy for TIMIT Dataset with k-nearest neighborhood classifier (KNN). The Deeper Long Short-Term Memory (LSTM) Networks structure for the prediction of gender from an audio dataset was described in the paper [16]. This proposed method produced high accuracy of 98.4% of gender detection. In [17], the authors proposed a semi-supervised algorithm, named iCST-Voting, for the gender detection from the audio which is assigned as the most popular self-labeled algorithm. They achieved gender detection accuracy at the level of 98.42%. In the paper of [18] a two level GMM classifier was applied to detect age and gender. The classification accuracy on gender and age recognition was 97.5%. Maka et al. [19] used 630 speakers, 438 males and 192 females in their experiments for the gender identification problem in different acoustical environments (indoor and outdoor auditory scenes). In their results, they found out that non-linear smoothing increases the classification accuracy by 2% and the recognition accuracy obtained was 99.4%.

There have also been attempts at applying both the x-vector and LSTM framework to the age estimation task. In [4], the authors have trained a LSTM-based system that has been shown to outperform the i-vector baseline on short-duration speech segments on NIST SRE 2010 dataset [20]. Another research group in [21] has developed a method based on x-vectors, which showed a mean absolute error (MAE) of 4.92 years. The implementation of the i-vector system on the same dataset enabled the MAE of 5.82, which is definitely lower.

The work in [22] describes a DNN implementation for a joint height and age estimation system. Their results for age estimation are 0.6 years in terms of root mean square error (RMSE), 7.60 and 8.63 years for male and female using the TIMIT dataset [23]. Finally, in the latest paper from 2020 [24], the authors propose a feature-engineering based support vector regression system and achieve a state-of-the-art results on the TIMIT dataset, with a mean absolute error (MAE) of 5.2 for males and 5.6 years for female.

In this paper, we present the DNN-based approach, enhancing with a multi-stage transfer learning schemes to detect gender and estimate the age of a speaker.

## 2. Data

To conduct the experiments we used 3 open-source, popular speech datasets: VoxCeleb1, Common Voice, and TIMIT. The first dataset was used to pre-train the speaker embedder. The remaining two were used for the experiments regarding age estimation and gender classification. Regardless of the original format and sampling frequency, all the data in each of the datasets is converted to 16 bit PCM wav format and downsampled to 16 kHz.

### 2.1. Voxceleb 1

VoxCeleb1 [25] is a large-scale audio-visual dataset published in 2017. It contains over 100,000 recordings from 1251 celebrities which correspond to over 300 h of data. The dataset was created primarily for the purpose of accelerating research in the field of speaker identification and verification. The database is gender-balanced. This dataset was used in transfer learning schemes to pre-train the speaker embedder network. It comes as 16-bit PCM wav files with a sampling rate of 16 kHz.

### 2.2. Common Voice

**Figure 1:**
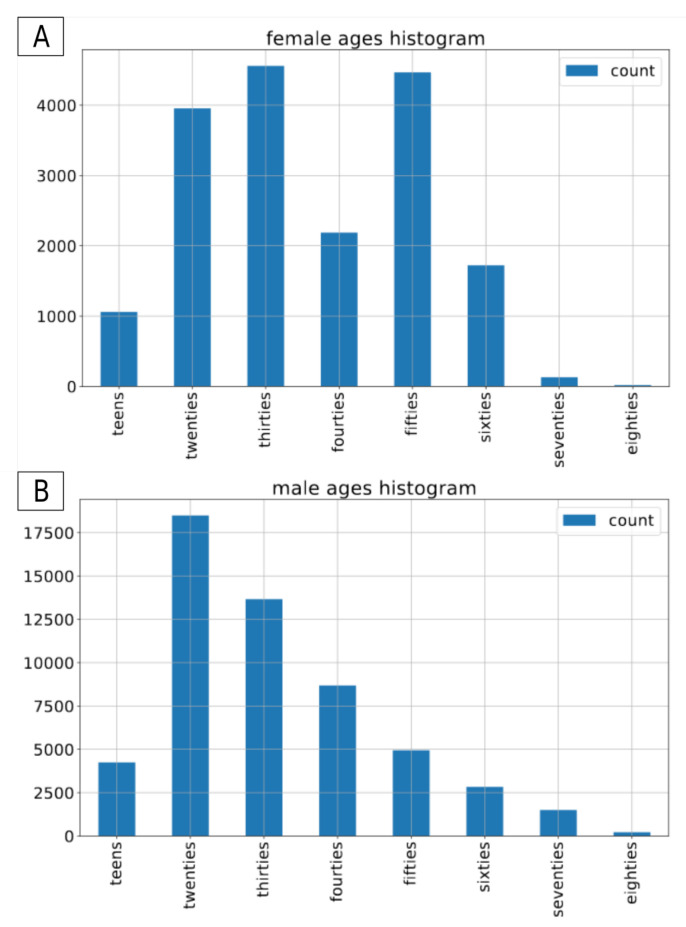
Common Voice: age distribution by gender in the train dataset. (**A**) female speakers, (**B**) for male speakers.

**Figure 2:**
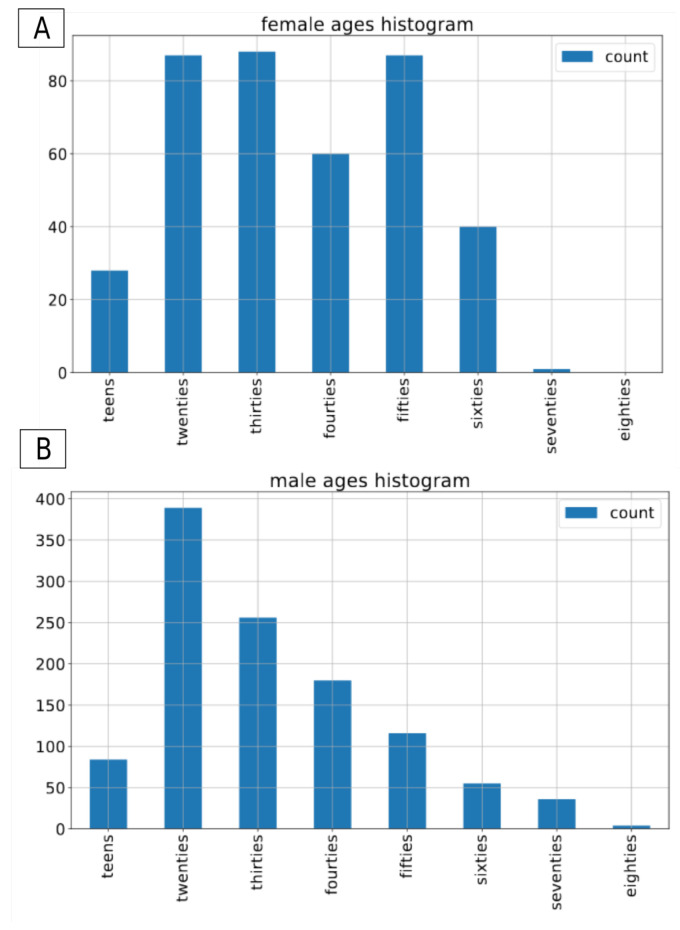
Common Voice: age distribution by gender in the validation dataset. (**A**) for female speakers, (**B**) for male speakers.

**Figure 3:**
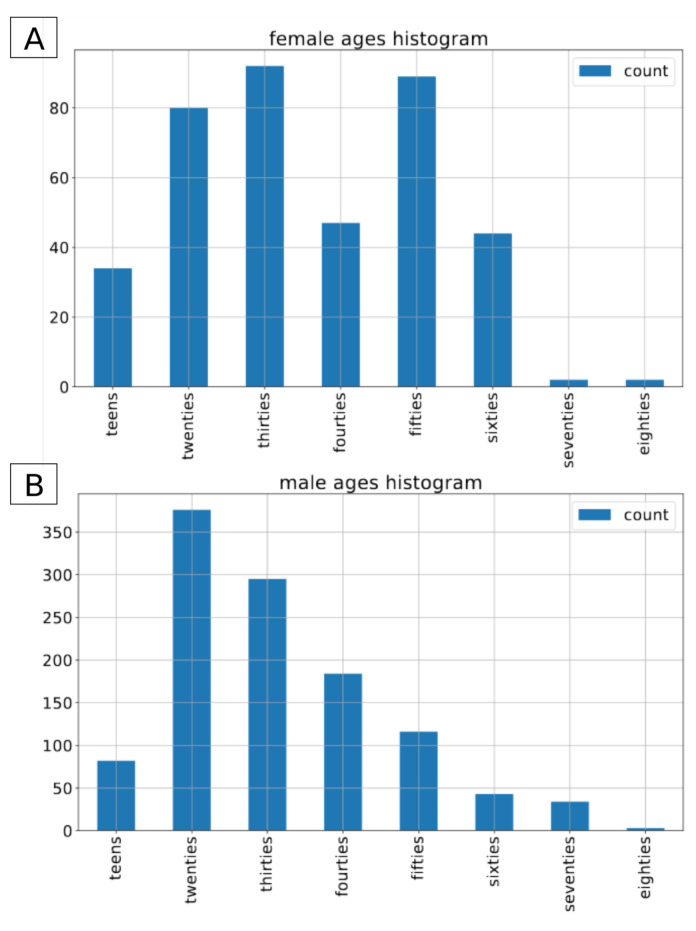
Common Voice: age distribution by gender in the test. (**A**) for female speakers, (**B**) for male speakers.

The Common Voice dataset is the largest open-source, multilingual dataset. It contains more than 2500 h of transcribed speech in 40 languages [26]. On top of the audio recordings and the corresponding transcriptions, it also contains voluntary metadata about the speaker, such as age group (teens, twenties, ..., eighties), gender (female, male, other) and accent. For the sake of this project we have decided to use the subset of the english part of the CommonVoice dataset [27]. To conduct the experiments we included only those recordings that contain metadata about both gender and age of the speaker. The recordings with label other for gender were excluded from this experiment. In total, we applied approximately 80 h of data in the train set, and 1.5 h of data in both validation and test sets. In terms of number of recordings, it consists of 54,593 male and 18,099 female recordings in the training set, 1120 and 391 male/female recordings in the validation set and 1133/390 male/female recordings in the test set. As there is no information about speakers’ labels in the version of dataset we used, it is not possible to determine the exact number of speakers in both gender groups. The original recordings come in an mp3 format with a sampling rate of 44,100 Hz. Figure 1, Figure 2 and Figure 3 present the distribution of recordings in different age groups in the training, validation, and test set, respectively.

### 2.3. Timit

The DARPA-TIMIT dataset [28] contains recordings of 630 speakers from 8 different English dialects. It contains rich metadata about each speaker, including gender (male/female), exact age (exact birth date and recording date), and accent. For training and validation, we applied a random TRAIN-TEST split on the default TRAIN subset of the data. That included 3.5 h of train data and 0.5 an hour of validation data. The test set was applied for final examination. That contained 1500 utterances and the length of recordings were 1.5 h.

**Figure 4:**
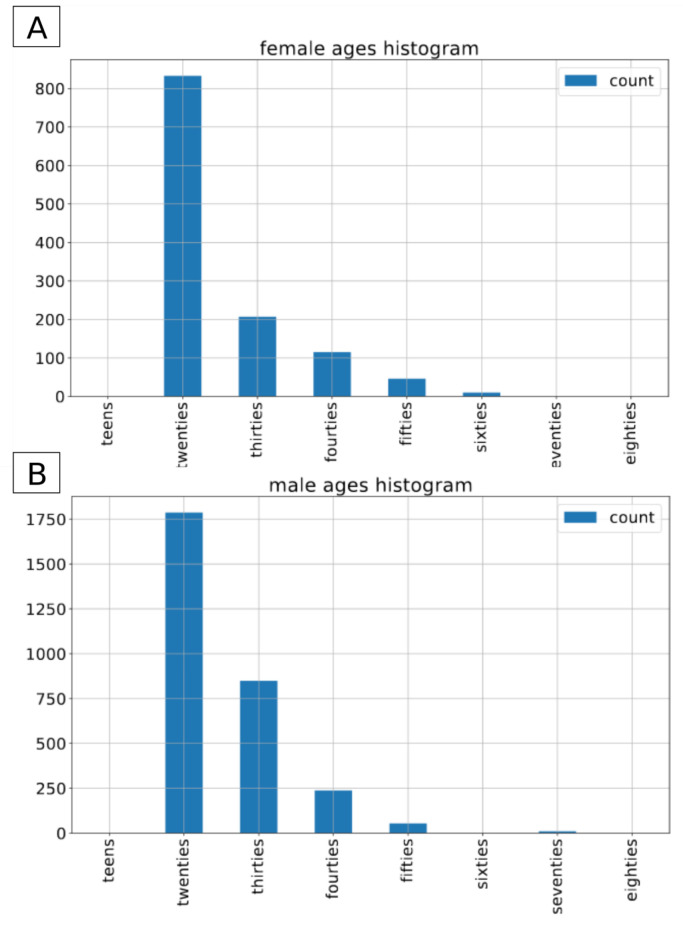
TIMIT: age distribution by gender in the train dataset. (**A**) female speakers, (**B**) male speakers.

**Figure 5:**
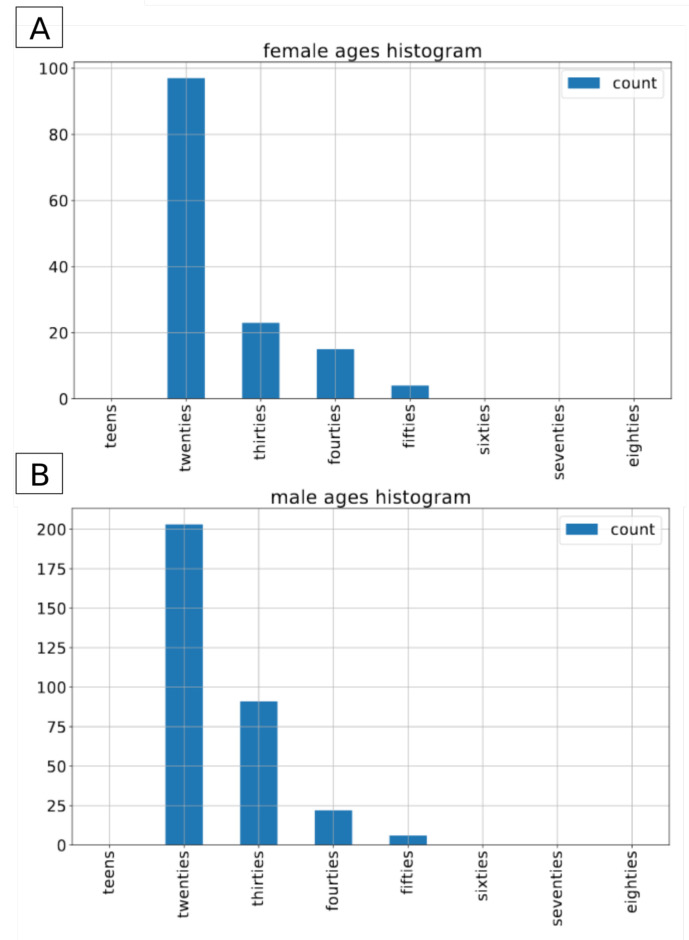
TIMIT: age distribution by gender in the validation dataset. (**A**) female speakers, (**B**) male speakers.

**Figure 6:**
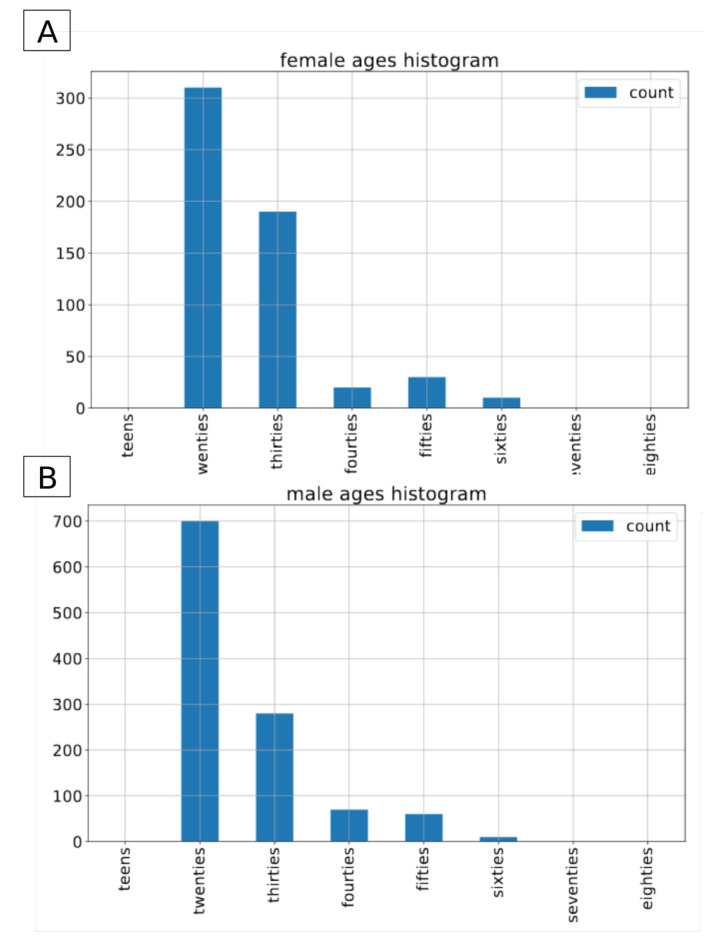
TIMIT: age distribution by gender in the test. (**A**) female speakers, (**B**) male speakers.

In terms of exact number of recordings, there are 2938 male and 1211 female recordings in the training set, 322 and 139 male/female recordings in the validation set and 1120/560 male/female recordings in the test set. The recordings are stored in the wav format and were recorder with a sampling rate of 16,000 Hz. For training and validation, a random TRAIN–TEST split is performed on the default TRAIN subset of the data, which corresponds to roughly 3.5 h of train data and 0.5 an hour of validation data. Figure 4, Figure 5 and Figure 6 present the distribution of data in the original test set as well as in randomly split train and validation sets.

## 3. Methods

**Figure 7:**
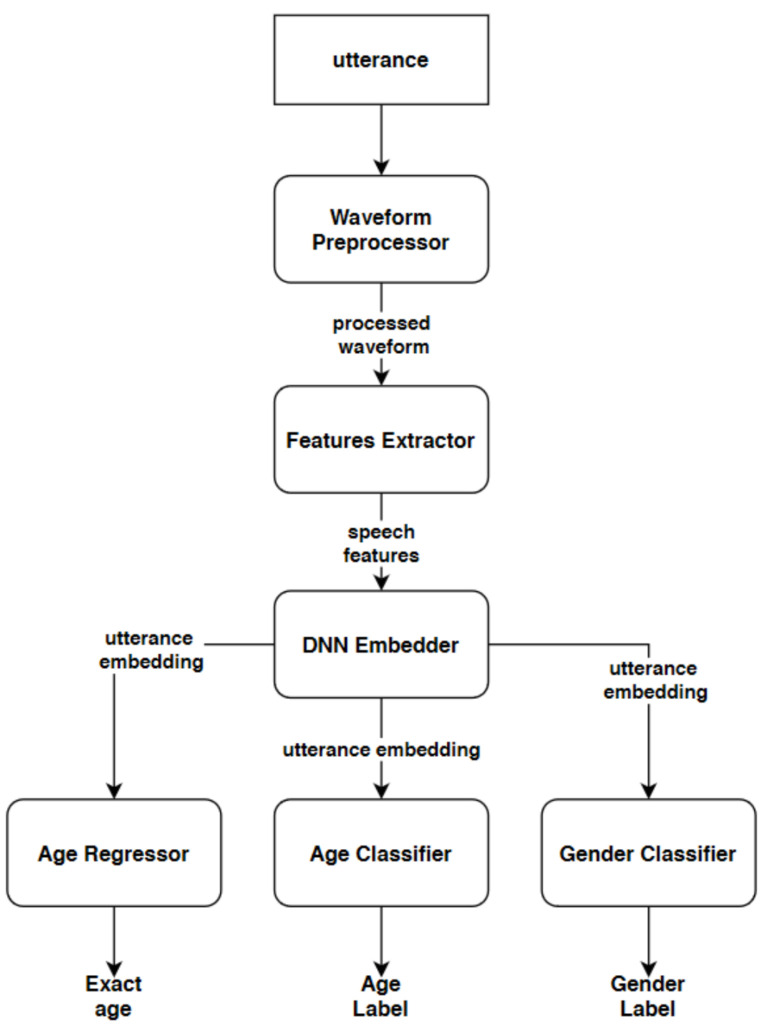
High-level representation of the system for joint age estimation and gender classification.

Figure 7 presents a high-level representation of the proposed system. Three different approaches are explored. Every system uses similar front-end classification/regressions modules, which differ in size depending on the embedder architecture used and, consequently embedding size. These are descirbed in more details in Section 3.2.

The first implemented system is the baseline x-vector solution introduced in [21], adapted for the joint age and gender prediction. The adaptation involves replacing layers 7–8 from the original paper with a more modular configuration of 2 separated classifiers and a regressor to allow for joint age/gender prediction. It features Voice Activity Detection (VAD) and a 5 s long random crop in the waveform preprocessing stage and 23-dimensional MFCCs with Cepstral Mean Normalization over a sliding windows of 3 s as input features. For the embedder, a vanilla TDNN is used, which we describe in more in details in Section 3.1.1 [8].

The second system is our proposition at extending the baseline version. Instead of 23-MFCCs, it uses a 30-dimensional MFCC features. Instead of Cepstral Mean Normalization, which experimentally has been shown to perform poorly for short utternaces in the TIMIT datas, we apply decibel-relative-to-full scale normalization to the level of −30 dB to the cropped waveforms before feature extraction.The idea to use this normalization method comes from in [10], where the authors used it in a d-vector based speaker verification system. Another major difference is the embedder architecture. It has been shown that deeper and more modern architectures such as TDNN-F or ResNet outperform the shallow TDNN-based embedder in the task of speaker verification [7]. Inspired by those result, we applied embedder that consists of a deep, residual convolutional architecture, described in more deatils in Section 3.1.2.

The third explored system differs heavily from the first two. First of all, instead of the x-vector embedder, it uses d-vector architecture introduced in [10]. The d-vector approach differs from x-vector in the way the utterance embedding is generated—instead of a deep convolutional network, a multilayer-LSTM RNN network is used, and the output of the last hidden unit is used as embedding. We present more details on this embedder architecture in Section 3.1.3. The second difference comes from the way the whole system is trained. While the first two systems train the embedder in an end-to-end manner alongside the front-end modules, the third system uses a pretrained embedder shared by the authors (which has been trained for speaker verification on LibriSpeech, VoxCeleb1 and VoxCeleb2 datasets) [29] and freezes its weights, which means that in this system only the front-end modules are trained. The motivation for such approach is the vast amount of computational power that would be required to train such system and the fundamental differences in the data processing pipelines and training scheme between that system and the remaining two.

**Figure 8:**
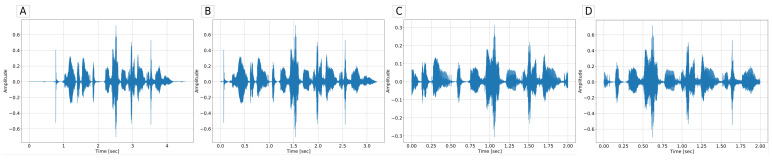
Different waveform preprocessor stages illustarted. (**A**) unprocessed waveform, (**B**) waveform after VAD, (**C**) waveform after random cropping 2 s of the VAD output, (**D**) applying dBFS normalization to the level of −30 dB to the output of stage C.

The effects of subsequent preprocessing stages mentioned earlier are shown in Figure 8.

### 3.1. Embedder Architectures

#### 3.1.1. Time Delay Neural Network Based X-Vector Embedder

The first explored embedder consists of a stack of time-delay neural networks layers that are responsible for capturing local context, followed by a statistics pooling layer, which aggregates outputs of the TDNN layers [8,21,30]. The statistics vector is then passed through a fully connected layer to form the final embedding. The TDNN layer has been implemented as a 1-D dilated convolution [31]. The detailed network architecture has been shown in Table 1.

#### 3.1.2. Quartznet X-Vector Embedder

The second flavor of the x-vector embedder we propose differs from the one described in Section 3.1.1 mainly in the architecture of the layers that precedes the statistics pooling layer. The quartznet x-vector embedder uses the quartznet architecture for the task of end-to-end speech recognition [32]. The network is composed of several blocks with residual connections between them. Each block is composed of one or more 1D convolution, batch normalization, and ReLU layers. The exact architecture that has been used throughout this work is shown in Table 2.

#### 3.1.3. D-Vector Embedder

The d-vector architecture used in this work has been introduced in [10] for the task of speaker verification. Although the goal of d-vector based embedder is the same as of the x-vector architecture, that is, to embed a variable-length utterance into a fixed size vector, the principle is much different. Instead of statistical pooling layers that aggregate information across the global context, the d-vector uses a multilayer-LSTM network [33] with a linear projection at the final layer to summarize the utterance. What is unique about the contribution of the authors at [10] is the training procedure, especially the introduction of the so-called *Generalized End-to-End* training. The network architecture itself is shown in Table 3.

### 3.2. Front-End Modules

As shown already in Figure 7, there are 3 additional neural networks on top of the embedder: binary classifier for gender classification, multiclassifier for helper age group classification, and regressor for age estimation. Due to the modular design of the presented system, embedder network can be trained separately from the classifiers/regressor and this feature has been proved very useful in the experiment where the embedder has been separately pretrained on the VoxCeleb dataset and then jointly fine-tuned on Common Voice and TIMIT for the gender/age classification task. The exact architecture of these front-end modules is presented in Table 4.

## 4. Results

We have performed experiments with three different methods of data processing and network architectures, introduced in previous sections. Each system presented in this section has been trained in three different ways:

- only on the TIMIT train dataset,
- only on the Common Voice train dataset, or
- pretrained on the Common Voice train dataset and then fine-tuned on the TIMIT train dataset.

The reasoning behind such training scheme is the attempt to leverage transfer learning. The Common Voice dataset used in this work is much larger then the TIMIT train dataset with more then 80 h of data as compared to approximately 3.5 h in the case of TIMIT. We present the results of these experiments in Section 4.1, Section 4.2 and Section 4.3. We also present one additional experiment, where an additional pretraining of embedder with a speaker identification task on the VoxCeleb dataset is performed. The results for this are shown in Section 4.4.

One important fact to note about the Common Voice dataset is that it does not contain speaker labels. In other words, it is entirely possible and perhaps highly likely, that speakers overlap between train, validation, and test datasets. As such, the classification results on the Common Voice dataset are presented mostly to show the performance of the system trained solely on the TIMIT dataset as well as to show one of negative features of transfer learning—so-called catastrophic forgetting [34], a phenomenon where a fine-tuned system no longer performs equally well on the original dataset.

The results are presented according to the following metrics: Mean Absolute Error (MAE), Root Mean Square Error (RMSE), Accuracy, and F1 score. The waveform processing and features extraction details for the baseline and QuartzNet-based systems are shown in the Table 5 and Table 6. As the d-vector-based system uses a neural network as a feature extractor, the details of processing for that system are not presented here.

### 4.1. Baseline Tdnn X-Vector System

The results of the baseline system achieved on the TIMIT test dataset are shown in Table 7. The hyperparameters of the baseline system strictly follow the baseline implementation proposed in [21]. The performance on the gender classification task on the Common Voice dataset with a baseline x-vector embedder is presented in Table 8. The age estimation RMSE of presented approach is 8.44 and 7.96 years for female and male speakers is comparable to the results reported by the authors at [22], with 8.63 and 7.60 years female/male. However, the network does not react well to the attempts of using transfer learning—a system pre-trained on Common Voice actually offers worse results then the system with no pre-training. One possible reason for such behaviour may be the usage of Cepstral Mean Normalization over a sliding window—the difference in lengths of recordings between Common Voice and TIMIT datasets, and the fact that the recordings in the TIMIT dataset are overall quite short (below 3 s on average), may lead to a mismatch in the estimates and conversely in the degradation of results. In fact we also found experimentally that usage of the Cepstral Mean Normalization in the second proposed architecture lead to worse results then different normalization techniques.

### 4.2. Quartznet-Based X-Vector System

The results of the QuartzNet-based system are shown in the Table 9 and 13. As we have found experimentally that the usage of CMVN lead to worse results, we decided on different normalization technique for this system. In particular, we decided to make use of the dbFS normalization used in the d-vector system [10] and apply it to the quartznet-based x-vector system. The QuartzNet-based system performs worse then baseline when the amount of data is low (trained only on TIMIT), which should not be a surprise given the size of the network it uses. However, it achieves a significant performance gain in the transfer learning scenario, clearly outperforming the baseline system when pretrained on the Common Voice dataset. On the TIMIT test set it achieves RMSE of 7.91 and 7.37 years for female/male and MAE of 5.2 and 5.37 years female/male. the results in terms of MAE are comparable with the state-of-the-art age estimation results of 4.23 and 5.78 female/male on the NIST SRE08 dataset which were published in [21]. Note, however, that the lengths of utterances in that dataset are much higher then of those in the TIMIT dataset and the authors report much worse results on shorter test segments. These results are also on-par with those recently published in [24] (5.6 and 5.2 MAE female/male) without using any hand-engineered features and relying solely on the low-level signal representation.

In terms of gender classification, a similar pattern as with age estimation can be seen. While on average the accuracy of gender classification is comparable to accuracy achieved with a baseline system, the performance tends to be worse when the amount of training is limited, which again, is expected due to the size of the network. This phenomenon can be seen comparing accuracy of systems trained only on TIMIT train dataset, shown in Table 8 and Table 13.

### 4.3. D-Vector Embedder-Based System

The D-vector embedder-based system uses the embedder network as feature extractor and does not update its weights during the training. This means that the only trainable parameters are located in the classifiers/regressor MLPs and the overall number of weights that needs to be optimized is lower then in the case of the end-to-end pipelines of baseline and quartznet systems by an order of magnitude. The complete pipeline of this approach was as published in [10], while the results on TIMIT and Common Voice datasets are shown in Table 10 and Table 11.

This system offers the best and the most robust results in terms of RMSE for age estimation and accuracy for gender classification. The performance of the systems when trained only on the TIMIT train dataset is already comparable to that of the baseline x-vector system in terms of age estimation and better in terms of gender classification accuracy. However, this system benefits from pre-training the classifiers and regressor on the Common Voice dataset, as it yields an improvement of 0.56 years RMSE when compared to the system trained without the pre-training. Furthermore, the performance on the gender classification task is the best out of the proposed solutions with 96.80% accuracy on the Common Voice test set in a scenario without any Common Voice data in the training, 99.40% on the TIMIT test set with no TIMIT data in training and 99.60% when trained on TIMIT train set or fine-tuned on it.

### 4.4. Pre-Training X-Vector Embedder on Voxceleb 1

Encouraged by the results achieved with the QuartzNet architecture and given how well it reacted to pretraining on Common Voice dataset, we decided to experiment with one extra pretraining step. In this pipeline, the quartznet-based embedder network (but only the embedder) receives an extra training step—it is pretrained on the VoxCeleb 1 dataset with the goal of speaker identification. The pretraining is performed using a MultiClassifier network as a frontend module with number of output classes equal to number of speakers in the VoxCeleb1 dataset, which is 1211. After training on VoxCeleb, the frontend module is discarded and only the pretrained embedder is used in further training steps. The hypothesis for this step is that the speaker’s identity contains also information about both speaker’s gender and age. The results obtained with this pipeline are shown in Table 12. The results seems to confirm the assumption—in every scenario, the age estimation results improved with regards to the same system without the VoxCeleb pre-training (shown in the Table 9 and Table 13) when MAE metric is considered (also the RMSE metric improved or remained the same). On top of that, the gender classification accuracy is competitive with the results achieved by the d-vector system, shown in Table 10. This results are also the best in terms of MAE out of all proposed solution and better then the current state-of-the-art results shown in [24] by 0.31 and 0.08 MAE for female and male speakers, respectively.

## 5. Conclusions

In this paper, we explored different neural network architectures, transfer learning schemes, and usage of multitask learning in the context of age estimation and gender classification from speech signals. In particular, we implemented baseline and extended x-vector based utterance embedder as well as a d-vector based system. The proposed transfer learning schemes, including pretraining systems on the Common Voice dataset as well as an additional embedder pre-training on VoxCeleb dataset, yielded consecutive performance gains in all scenarios, except for the baseline system. The results presented in this work confirm that these deep learning approaches are effective at estimating speaker’s age and gender. In terms of age estimation, the proposed system with two-staged transfer learning scheme and a QuartzNet embedder achieved new state-of-the-art result on the TIMIT dataset, with a MAE of 5.12 years for male, 5.29 years for female speakers, and RMSE of 7.24 and 8.12 years for male and female speakers respectively. Comparing to the results already published in the literature (Table 14), our algorithm overcomes existing solutions published in the literature. In terms of gender classification, the d-vector-based system achieved a robustly high performance with accuracies varying from 96.8% to 99.6% depending on the training and testing datasets. The highest result was achieved when the Common Voice dataset was used for training, the algorithm was further fine-tuned on TIMIT dataset, what enabled the classification accuracy at the level of 99.6% for gender recognition. The female accuracy with proposed preprocessing and classification methods was slightly ( 0.1%) worse than the male recognition. This result also overcomes the existing methods published in the literature so far (Table 15). An additional appeal of the d-vector system is that it only contains trainable weights in the front-end modules, reducing amount of data needed to acquire satisfying performance, overfitting and training time. In overall, the accuracy detection of female was lover for all the presented methods in this paper. There are some limitations to this study that should be kept in mind when interpreting the findings. The transfer learning approach, we used built on the information that already exist in pre-trained models. Secondly, the databases used for the purpose of this research are not meant to be fully representative of all the potential diversity in human voices. In the end, the aim of this research was to capture the signals’ features that were diverse enough to make a meaningful comparisons about the way these types of system learn about gender recognition and age estimation. This research provided insights into the nature and limitations of the implemented types of machine learning models. In general, it is important to train the models on the data to capture the diversity of human characteristics, they will encounter in real-world contexts.

For the future, it would be interesting to explore more recently proposed architectures, like wav2vec2.0 [35] to obtain the embeddings from raw waveforms. It would also be beneficial to explore multi-language or language independent extension of the proposed methodology, for example by using other languages available in the CommonVoice dataset or the recently published MLS dataset [36].

## Figures and Tables

**Table 1:** Baseline architecture summary [21].

Layer	Layer Context	Size
TDNN1	[0]	400
TDNN2	[−2,0,2]	400
TDNN3	[−3,0,3]	400
TDNN4	[0]	400
Stats pooling (Mean + STD)	[0,T)	1500 + 1500
Dense + ReLu	*T*	400

**Table 2:** Quartznet architecture summary [21].

Block Name	Kernel Length	Repeats	Residual	Size
Input	3	1	True	512
$Block_{1}$	5	2	True	512
$Block_{2}$	7	2	True	512
$Block_{3}$	9	2	True	512
Final	1	1	False	512
pooling (Mean + STD)	−	1	False	1500 + 1500
Dense + BatchNorm + ReLu	−	1	False	512
Dense + BatchNorm + ReLu	−	1	False	512

**Table 3:** Dvector network architecture summary [10].

Layer	Layer Size
LSTM_1	256
LSTM_2	256
LSTM_3	256
Dense + ReLu	256

**Table 4:** The exact architectures of front-end modules.

**Binary Classifier Network for Gender Classification**		
Layer	Input Size	Output Size
Dense + ReLu + BatchNorm	embedding size	embedding size
Dense + Sigmoid	embedding size	1
**Multi Classifier Network for Age Group Classification or Speaker Identification**		
Layer	Input Size	Output Size
Dense + ReLu + BatchNorm	embedding size	embedding size
Dense + Logits	embedding size	number of classes
**Regressor Network for Age Estimation**		
Layer	Input Size	Output Size
Dense + ReLu + BatchNorm	embedding size	embedding size
Dense + Logits	embedding size	1

**Table 5:** Waveform processing details for baseline and QuartzNet-based systems.

System	Waveform Processing		
	VAD	Random Crop	dBFS Normalisation
Baseline	TRUE	5 s	-
Proposed with Quartznet	TRUE	5 s	−30 dBFS

**Table 6:** Features extraction details for baseline and QuartzNet-based systems.

System	Features Extraction							
	Type	No of Features	Low Cut-off Frequency	High Cut-off Frequency	Window Size	Window Step	Window	Post-Processing
Baseline	MFCC	23	40 Hz	8000 Hz	25 ms	10 ms	Hamming	Cepstral Mean Normalization, 3 s window
Proposed with Quartznet	MFCC	30	40 Hz	8000 Hz	25 ms	10 ms	Hamming	-

**Table 7:** Results on the TIMIT test dataset with a baseline x-vector embedder.

Group	Trained on	Fine-Tuned on	Gender Results	Age Results	
	TIMIT train	-	Accuracy	MAE	RMSE
All			98.30%	5.73	8.12
Female			95.70%	5.84	8.44
Male			99.60%	5.67	7.96
	Common Voice Train	-	Accuracy	MAE	RMSE
All			98.80%	7.43	10.23
Female			96.80%	7.65	10.46
Male			99.80%	7.32	10.11
	Common Voice Train	TIMIT train	Accuracy	MAE	RMSE
All			98.80%	5.72	8.25
Female			96.80%	5.64	8.42
Male			99.80%	5.77	8.16

**Table 8:** Results on the Common Voice test set with a baseline x-vector embedder.

Group	Trained on	Fine-Tuned on	Gender Results	Age Results
	TIMIT train	-	Accuracy	Weighted_F1
All			91.10%	24%
Female			75.40%	23%
Male			96.60%	24%
	Common Voice Train	-	Accuracy	Weighted_F1
All			98.00%	68%
Female			95.40%	71%
Male			98.90%	66%
	Common Voice Train	TIMIT train	Accuracy	Weighted_F1
All			94.20%	31%
Female			90.50%	28%
Male			95.50%	32%

**Table 9:** Results on the TIMIT est dataset with a Quartznet-based x-vector embedder and volume normalization.

Group	Trained on	Fine-Tuned on	Gender Results	Age Results	
	TIMIT train	-	Accuracy	MAE	RMSE
All			98.30%	5.98	8.47
Female			97.00%	6.28	9.42
Male			98.90%	5.83	7.96
	Common Voice Train	-	Accuracy	MAE	RMSE
All			97.70%	7.97	10.32
Female			95.50%	7.06	9.77
Male			98.80%	8.42	10.59
	Common Voice Train	TIMIT train	Accuracy	MAE	RMSE
All			98.90%	5.31	7.55
Female			97.10%	5.2	7.91
Male			99.80%	5.37	7.37

**Table 10:** Results on the TIMIT test dataset with a pre-trained d-vector embedder as features source.

Group	Trained on	Fine-Tuned on	Gender Results	Age Results	
	TIMIT train	-	Accuracy	MAE	RMSE
All			99.60%	5.93	8.08
Female			99.50%	6.15	8.81
Male			99.60%	5.83	7.68
	Common Voice Train	-	Accuracy	MAE	RMSE
All			99.40%	10.5	13.48
Female			99.10%	12.17	14.97
Male			99.60%	9.67	12.67
	Common Voice Train	TIMIT train	Accuracy	MAE	RMSE
All			99.60%	5.46	7.52
Female			99.50%	5.75	8.2
Male			99.60%	5.32	7.16

**Table 11:** Results on the Common Voice datatest set with a pretrained d-vector embedder as features source.

Group	Trained on	Fine-Tuned on	Gender Results	Age Results
	TIMIT train	-	Accuracy	Weighted_F1
All			96.80%	27%
Female			93.80%	19%
Male			97.80%	29%
	Common Voice Train	-	Accuracy	Weighted_F1
All			98.20%	65%
Female			96.70%	66%
Male			98.60%	64%
	Common Voice Train	TIMIT train	Accuracy	Weighted_F1
All			96.90%	35%
Female			93.30%	29%
Male			98.20%	37%

**Table 12:** Results on the TIMIT test dataset with a Quartznet-based x-vector embedder with dbFS normalization and additional embedder pre-training on the VoxCeleb 1 dataset.

Group	Pretrained on 1	Fine-Tuned on 1	Fine-Tuned on 2	Gender Results	Age Results	
	VoxCeleb1	TIMIT train	-	Accuracy	MAE	RMSE
All				98.50%	5.37	7.74
Female				96.40%	5.65	8.53
Male				99.50%	5.23	7.31
	VoxCeleb1	Common Voice train	-	Accuracy	MAE	RMSE
All				98.50%	7.76	10.05
Female				97.00%	8,01	10.58
Male				99.30%	7.64	9.77
	VoxCeleb1	Common Voice train	TIMIT train	Accuracy	MAE	RMSE
All				99.60%	5.18	7.54
Female				98.80%	5.29	8.12
Male				100.00%	5.12	7.24

**Table 13:** Results on the Common Voice test set with a Quartznet-based x-vector embedder and volume normalization.

Group	Trained on	Fine-Tuned on	Gender Results	Age Results
	TIMIT train	-	Accuracy	Weighted_F1
All			88.00%	19%
Female			57.90%	19%
Male			98.30%	20%
	Common Voice Train	-	Accuracy	Weighted_F1
All			99.60%	93%
Female			99.00%	94%
Male			99.80%	93%
	Common Voice Train	TIMIT train	Accuracy	Weighted_F1
All			87.30%	25%
Female			51.80%	28%
Male			99.50%	25%

**Table 14:** Comparison of proposed system with existing ones for age estimation.

Published	Methods	Age Estimation (MAE)	Age Estimation (RMSE)	DataSet
[21]	x-vectors	4.92	-	NIST SRE 2010
[21]	x-vectors with baseline i-vector system	5.82	-	NIST SRE 2010
[22]	DNN initialization sheme	-	7.60 for male; 8.63 female	TIMIT
[24]	feature-engineering based support vector regression system	5.20 for male; 5.60 for female	-	TIMIT
(This paper) 1	x-vector with QuartzNet embedder and 2-stage transfer learning	5.12 for male; 5.29 for female	7.24 for male; 8.12 for female	TIMIT
(This paper) 2	d-vector feature extractor with frontend modules pretraining on Common Voice	5.32 for male; 5.75 for female	7.16 for male; 8.20 for female	TIMIT

**Table 15:** Comparison of proposed system with existing ones for gender recognition.

Published	Methods	Gender Recognition (Accuracy)
[11]	MLP	96.00%
[12]	MLP, GMM, vector quantization, learning vector quantization	96.40%
[13]	MLP	96.74%
[15]	k-NN, MLP	96.80%
[17]	GMM	97.50%
[14]	SVM	98.27%
[16]	LSTM	98.40%
[17]	iCST-Voting	98.42%
[19]	SVM	99.40%
(This paper) 1	x-vector with QuartzNet embedder and 2-stage transfer learning	99.60%
(This paper) 2	d-vector feature extractor with frontend modules pretraining on Common Voice	99.60%

## Data Availability

The data presented in this study are openly available in VoxCeleb1 at [37], the Common Voice dataset at [27] and The DARPA-TIMIT dataset [28].

## References

[1] Schuller B., Steidl S., Batliner A., Burkhardt F., Devillers L., MüLler C., Narayanan S. (2013). Paralinguistics in speech and language—State-of-the-art and the challenge. Comput. Speech Lang..

[2] Panek D., Skalski A., Gajda J., Tadeusiewicz R. (2015). Acoustic analysis assessment in speech pathology detection. Int. J. Appl. Math. Comput. Sci..

[3] Techmo. https://www.techmo.pl.

[4] Zazo R., Sankar Nidadavolu P., Chen N., Gonzalez-Rodriguez J., Dehak N. (2018). Age Estimation in Short Speech Utterances Based on LSTM Recurrent Neural Networks. IEEE Access.

[5] Mahmoodi D., Marvi H., Taghizadeh M., Soleimani A., Razzazi F., Mahmoodi M. Age Estimation Based on Speech Features and Support Vector Machine. Proceedings of the 2011 3rd Computer Science and Electronic Engineering Conference (CEEC).

[6] Dehak N., Kenny P.J., Dehak R., Dumouchel P., Ouellet P. (2011). Front-End Factor Analysis for Speaker Verification. IEEE Trans. Audio Speech Lang..

[7] Villalba J., Chen N., Snyder D., Garcia-Romero D., McCree A., Sell G., Borgstrom J., Richardson F., Shon S., Grondin F. State-of-the-Art Speaker Recognition for Telephone and Video Speech: The JHU-MIT Submission for NIST SRE18. Proceedings of the INTERSPEECH 2019.

[8] Snyder D., Garcia-Romero D., Sell G., Povey D., Khudanpur S. X-vectors: Robust dnn embeddings for speaker recognition. Proceedings of the 2018 IEEE International Conference on Acoustics, Speech and Signal Processing (ICASSP).

[9] McLaren M., Lawson A., Ferrer L., Castan D., Graciarena M. The speakers in the wild speaker recognition challenge plan. Proceedings of the Interspeech 2016 Special Session.

[10] Wan L., Wang Q., Papir A., Moreno I.L. Generalized end-to-end loss for speaker verification. Proceedings of the 2018 IEEE International Conference on Acoustics, Speech and Signal Processing (ICASSP).

[11] Jasuja L., Rasool A., Hajela G. Voice Gender Recognizer Recognition of Gender from Voice using Deep Neural Networks. Proceedings of the 2020 International Conference on Smart Electronics and Communication (ICOSEC).

[12] Djemili R., Bourouba H., Korba M.C.A. A speech signal based gender identification system using four classifiers. Proceedings of the 2012 International Conference on Multimedia Computing and Systems.

[13] Buyukyilmaz M., Cibikdiken A.O. Voice gender recognition using deep learning. Proceedings of the 2016 International Conference on Modeling, Simulation and Optimization Technologies and Applications.

[14] Alhussein M., Ali Z., Imran M., Abdul W. (2016). Automatic gender detection based on characteristics of vocal folds for mobile healthcare system. Mob. Inf. Syst..

[15] Uddin M.A., Hossain M.S., Pathan R.K., Biswas M. Gender Recognition from Human Voice using Multi-Layer Architecture. Proceedings of the 2020 International Conference on INnovations in Intelligent SysTems and Applications (INISTA).

[16] Ertam F. (2019). An effective gender recognition approach using voice data via deeper LSTM networks. Appl. Acoust..

[17] Livieris I.E., Pintelas E., Pintelas P. (2019). Gender recognition by voice using an improved self-labeled algorithm. Mach. Learn. Knowl. Extr..

[18] Pribil J., Pribilova A., Matousek J. (2017). GMM-based speaker age and gender classification in Czech and Slovak. J. Electr. Eng..

[19] Maka T., Dziurzanski P. An analysis of the influence of acoustical adverse conditions on speaker gender identification. Proceedings of the XXII Annual Pacific Voice Conference (PVC).

[20] Craig G., Alvin M., David G., Linda B., Kevin W. 2010 NIST Speaker Recognition Evaluation Test Set. https://catalog.ldc.upenn.edu/LDC2017S06.

[21] Ghahremani P., Nidadavolu P.S., Chen N., Villalba J., Povey D., Khudanpur S., Dehak N. End-to-end Deep Neural Network Age Estimation. Proceedings of the Interspeech 2018.

[22] Kalluri S.B., Vijayasenan D., Ganapathy S. A Deep Neural Network Based End to End Model for Joint Height and Age Estimation from Short Duration Speech. Proceedings of the 2019 IEEE International Conference on Acoustics, Speech and Signal Processing (ICASSP).

[23] Garofolo J., Lamel L., Fisher W., Fiscus J., Pallett D., Dahlgren N., Zue V. (1992). TIMIT Acoustic-phonetic Continuous Speech Corpus. Linguist. Data Consort..

[24] Kalluri S.B., Vijayasenan D., Ganapathy S. (2020). Automatic speaker profiling from short duration speech data. Speech Commun..

[25] Nagrani A., Chung J.S., Zisserman A. (2017). Voxceleb: A large-scale speaker identification dataset. arXiv.

[26] Ardila R., Branson M., Davis K., Henretty M., Kohler M., Meyer J., Morais R., Saunders L., Tyers F.M., Weber G. (2019). Common Voice: A Massively-Multilingual Speech Corpus. arXiv.

[27] Common Voice Database. https://www.kaggle.com/mozillaorg/common-voice.

[28] DARPA-TIMIT dataset. https://www.kaggle.com/mfekadu/darpa-timit-acousticphonetic-continuous-speech.

[29] Resemblyryzer. https://github.com/resemble-ai/Resemblyzer.

[30] Peddinti V., Povey D., Khudanpur S. A time delay neural network architecture for efficient modeling of long temporal contexts. Proceedings of the Sixteenth Annual Conference of the International Speech Communication Association.

[31] Waibel A., Hanazawa T., Hinton G., Shikano K., Lang K.J. (1989). Phoneme recognition using time-delay neural networks. IEEE Trans. Acoust. Speech Signal.

[32] Kriman S., Beliaev S., Ginsburg B., Huang J., Kuchaiev O., Lavrukhin V., Leary R., Li J., Zhang Y. Quartznet: Deep automatic speech recognition with 1d time-channel separable convolutions. Proceedings of the 2020 IEEE International Conference on Acoustics, Speech and Signal Processing (ICASSP).

[33] Sak H., Senior A.W., Beaufays F. (2014). Long short-term memory recurrent neural network architectures for large scale acoustic modeling. arXiv.

[34] Lee S.W., Kim J.H., Jun J., Ha J.W., Zhang B.T. Overcoming catastrophic forgetting by incremental moment matching. Proceedings of the Advances in neural information processing systems.

[35] Baevski A., Zhou H., rahman Mohamed A., Auli M. (2020). wav2vec 2.0: A Framework for Self-Supervised Learning of Speech Representations. arXiv.

[36] Pratap V., Xu Q., Sriram A., Synnaeve G., Collobert R. (2020). MLS: A Large-Scale Multilingual Dataset for Speech Research. arXiv.

[37] VoxCeleb1. https://www.robots.ox.ac.uk/~vgg/data/voxceleb/index.html#portfolio.

